# Limited developmental neurotoxicity from neonatal inhalation exposure to diesel exhaust particles in C57BL/6 mice

**DOI:** 10.1186/s12989-018-0287-8

**Published:** 2019-01-07

**Authors:** Keith Morris-Schaffer, Alyssa K. Merrill, Candace Wong, Katrina Jew, Marissa Sobolewski, Deborah A. Cory-Slechta

**Affiliations:** Department of Environmental Medicine, Box EHSC, University of Rochester Medical Center, Rochester, NY 14642 USA

## Abstract

**Background:**

Recent epidemiological studies indicate early-life exposure to pollution particulate is associated with adverse neurodevelopmental outcomes. The need is arising to evaluate the risks conferred by individual components and sources of air pollution to provide a framework for the regulation of the most relevant components for public health protection. Previous studies in rodent models have shown diesel particulate matter has neurotoxic potential and could be a health concern for neurodevelopment. The present study shows an evaluation of pathological and protracted behavioral alterations following neonatal exposure to aerosolized diesel exhaust particles (NIST SRM 1650b). The particular behavioral focus was on temporal control learning, a broad and fundamental cognitive domain in which reward delivery is contingent on a fixed interval schedule. For this purpose, C57BL/6 J mice were exposed to aerosolized NIST SRM 1650b, a well-characterized diesel particulate material, from postnatal days 4–7 and 10–13, for four hours per day. Pathological features, including glial fibrillary-acidic protein, myelin basic protein expression in the corpus callosum, and ventriculomegaly, as well as learning alterations were measured to determine the extent to which NIST SRM 1650b would induce developmental neurotoxicity.

**Results:**

Twenty-four hours following exposure significant increases in glial-fibrillary acidic protein (GFAP) in the corpus callosum and cortex of exposed male mice were present. Additionally, the body weights of juvenile and early adult diesel particle exposed males were lower than controls, although the difference was not statistically significant. No treatment-related differences in males or females on overall locomotor activity or temporal learning during adulthood were observed in response to diesel particulate exposure.

**Conclusion:**

While some sex and regional-specific pathological alterations in GFAP immunoreactivity suggestive of an inflammatory reaction to SRM 1650b were observed, the lack of protracted behavioral and pathological deficits suggests further clarity is needed on the developmental effects of diesel emissions prior to enacting regulatory guidelines.

**Electronic supplementary material:**

The online version of this article (10.1186/s12989-018-0287-8) contains supplementary material, which is available to authorized users.

## Background

Air pollution is a heterogeneous mixture of gases, volatile organic compounds, and particulates, all have the potential to influence the development of the central nervous system (CNS). The ultrafine fraction of pollution particulate (UFPs, < 0.1 μm) is considered to be the most toxic, as UFPs can achieve higher particle count concentrations and surface area at the same mass as the larger fractions, fine (< 2.5 μm) and coarse (< 10 μm) particulate matter [1]. UFPs are shown to deposit with high efficiency in the alveolar region of the lungs where they can pass through the lung epithelium and from there may become retained in the interstitium or translocate to the capillaries [2–4]. The UFPs retained in the lung have the potential to induce local inflammatory events that can trigger systemic alterations in secondary and tertiary messengers, chemokines, and cytokines which can adversely influence distal organs, such as the CNS. Alternatively UFPs have been shown to directly enter the CNS via the olfactory epithelium [5, 6]. Either through distal inflammatory pulmonary events that induce systemic inflammation or direct induction of damage to the CNS, UFPs can potentially influence CNS development [7–11], a critical period of neuro- and glia- genesis, cell migration, and cell differentiation.

Early-life exposure to pollution particulate is linked to adverse neurodevelopmental outcomes in humans, including hyperactivity, cognitive outcomes, and autism spectrum disorder (ASD). Increased exposure to elemental carbon from traffic sources during the first year of life was associated with an increase in hyperactivity score on the Behavioral Assessment for Children [12]. Another study in Boston found ambient black carbon exposure through early life was associated with decreases in visual and verbal learning on the Wide Range Assessment of Memory and Learning [13]. Several studies in Los Angeles have linked pre- and post- natal exposure to traffic-derived pollution particulate to an increased risk of developing ASD [14, 15]. These epidemiological studies have been complemented by developmental rodent models showing exposure to ambient fine and ultrafine particles can lead to neuroinflammation [8, 9, 16], white matter damage [10, 17], impulsivity [7], and cognitive dysfunction [18]. However, the exact constituent within ambient pollution particulate or its producers that may be contributing directly to adverse neurodevelopment outcomes is unclear. Determining which potential sources are major contributors to developmental neurotoxicity is critical for implementing a practical regulatory framework in mitigating the harm induced by exposure to pollution particulate.

One potential source that may contribute to the neurotoxic potential of pollution particulate is diesel exhaust particles. Diesel exhaust is a particular concern for public health as it is a substantial contributor to the ultrafine emission fraction on highways within the United States [19–21]. Off- and on-road diesel emissions have been estimated to contribute > 50% of the ambient black carbon within the Los Angeles and San Francisco Bay Area in 2010 [22]. Furthermore in Baltimore, diesel emissions were estimated to contribute 6.6–14.1% of the total fine particle matter concentration [19]. Diesel particulate matter has also been shown to be a potential contributor to pulmonary and cardiovascular toxicological outcomes in urban areas nationwide [19]. In Atlanta, diesel emissions have been linked to an increase in emergency department visits for asthma and wheezing [20]. The direct link between diesel particulate matter and effects on the human CNS is limited, although one study found acute exposure to high level of diesel exhaust particles altered electroencephalography readings in the frontal cortex of healthy volunteers [21].

The diesel particulate material used in this study was NIST SRM 1650b, a diesel particulate material generated in 1984 by direct injection four-cycle diesel engines within a dilution tube facility and collected directly from the heat exchangers [23]. NIST SRM 1650b is well-characterized, including known polycyclic aromatic hydrocarbons (PAH) [23], reactive metal content [24], as well as the relative ratio of organic and elemental carbon content [25]. Some of the characterized PAH found in NIST SRM 1650b are known to be neurotoxicants, including benzo[a]pyrene [26, 27] and fluoranthene [28]. NIST SRM 1650b’s characterized mean particle diameter is 0.18 μm with a strong peak in the ultrafine range [23], making it ideal in investigating the neurotoxic potential of diesel particulate matter. Overall NIST SRM 1650b can be considered a general representative of heavy equipment diesel emissions and is still currently used as a reference material for a variety of studies characterizing modern day particulate emissions [29–31]. Additionally, studies have shown NIST SRM diesel materials can recapitulate some rudimentary toxicological effects of modern diesel fuel, including in vitro ROS generation in a human cell line and in vivo pulmonary inflammatory cytokine increase [32, 33].

The toxicological consequences of NIST SRM 1650b, particularly with regards to pulmonary effects, have been previously explored through in vitro and in vivo studies. Direct application of NIST SRM 1650b to A549 type II epithelial cells increased the release of lactate dehydrogenase (LDH), a marker of cell damage, and DNA damage as measured by the Comet Assay [34]. Another study found the application of NIST SRM 1650b to human bronchial epithelial cells led to similar outcomes as diesel exhaust particulate from a modern European car, including a release of LDH at similar doses and an increase in granulocyte macrophage colony stimulating factor release [35]. A single nose-only inhalation exposure of BALB/c mice to 20 mg/m^3^ NIST SRM 1650b increased IL-6, CXCL1 expression [36], and heme oxygenase expression within the lung [37]. A paucity of research exists with regards to effects of NIST SRM 1650b specifically on the CNS, though one study found direct application of NIST SRM 1650b to human brain endothelial cells, an in vitro blood brain barrier model, decreased cell viability and increased ROS generation [38]. The current literature suggests potential mechanisms, both indirectly and directly, by which NIST SRM 1650b could potentially induce developmental neurotoxicity.

Given the relevance of diesel as a substantial contributor to pollution particulate within urban areas and the epidemiological evidence demonstrating ambient pollution particulate exposure is associated with adverse neurodevelopmental outcomes, it is useful from a regulatory perspective to evaluate whether diesel particle exposure alone can drive those outcomes. The purpose of the proposed study is to investigate if early-life exposure to diesel particulate matter at a high but human relevant-dose can invoke neurodevelopmental pathologies, including neuroinflammation, white matter disruption, ventriculomegaly, as well as protracted learning dysfunction. The exposure period is equivalent to the perinatal window in humans [30], a critical window of development within the CNS, including the formation of the blood-brain-barrier [39], gliagenesis [40], and gray matter growth [41, 42]. Previous studies have shown diesel particles can induce neuroinflammatory-like events, including disrupting the blood-brain-barrier integrity [43, 44], increasing inflammatory cytokine expression within the CNS [45, 46], and prime glial activation [47, 48], suggesting some plausibility the diesel particulate matter exposure during a vulnerable period can lead to protracted CNS dysfunction. The learning paradigm utilized in the present study is the fixed interval schedule, an effective assay at detecting the protracted learning effects of a wide-range of developmental environmental toxicants, including lead [49, 50], methylmercury [51, 52], and polychlorinated biphenyls [53, 54].

## Results

### Exposure

**Fig. 1:**
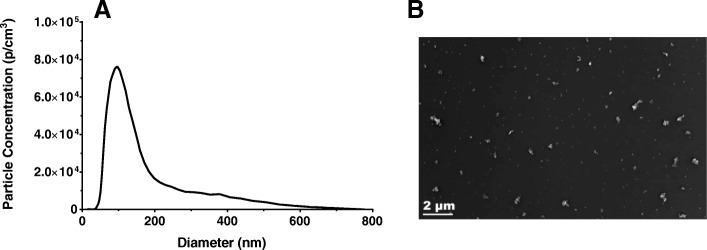
Exposure Characterization. Size distribution of the NIST SRM 1650b aerosol was produced with the ultrasonic nebulizer and measured with an electrostatic classifier with an example from one session shown (**a**)**.** TEM images of SRM aerosol particulate collected on a carbon-coated copper grid via electrostatic precipitation (**b**)

A sample size distribution of the particles collected during an exposure session is shown in Fig. 1a. The average count median diameter (CMD) across all exposure days was 105.8 ± 6.52 nm with an average GSD of 1.67 and the average mass concentration was 100 ± 0.013 μg/m^3^. The transmission electron microscopy (TEM) images of the collected aerosolized NIST SRM 1650b show a heterogeneous mixture of particles of different sizes and morphologies (Fig. 1b).

### Pathology

**Fig. 2:**
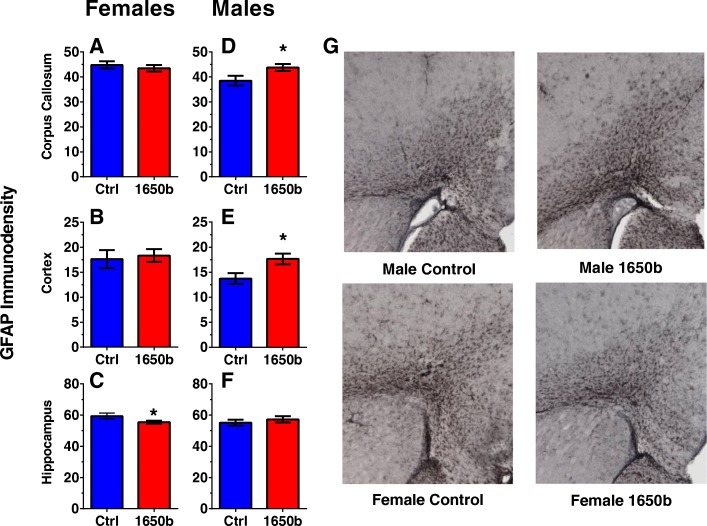
Regional GFAP immunoreactivity 24 h after exposure**.** Relative staining intensity of GFAP in the corpus callosum, cortex, and hippocampus respectively in females (**a, b, c**) and males (**d, e, f**)**.** *n* = 6 mice/sex/treatment group. Representative images of GFAP staining in females and males (**g**). Data are reported as group mean staining intensity for each treatment group ± SE

**Fig. 3:**
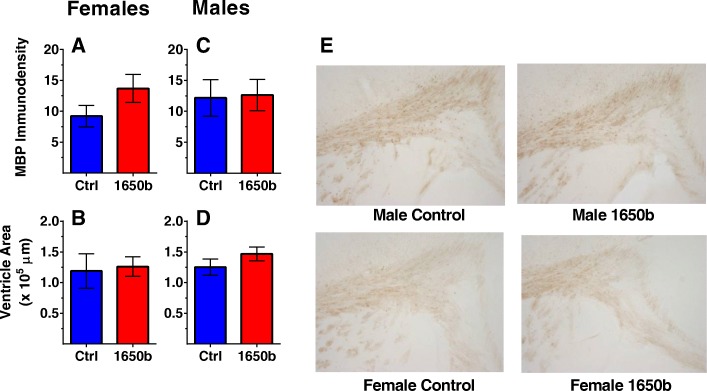
MBP immunoreactivity in the corpus callosum and lateral ventricle size 24 h after exposure. Relative staining intensity of MBP in the corpus callosum in females (**a**) and males (**c**)**.** Average lateral ventricle area in females (**b**) and males (**d**). *n* = 6 mice/sex/treatment group. Representative images of MBP staining in females and males (E). Data are reported as group mean staining intensity or ventricle area for each treatment group ± SE

Levels of GFAP immunoreactivity in the corpus callosum, frontal cortex, and hippocampus are shown for females (Fig. 2a-c) and males (Fig. 2d-f). MBP immunoreactivity in the corpus callosum and ventricle area size is shown for females (Fig. 3a, b) and males (Fig. 3c, d). There were no significant treatment-related GFAP immunostaining differences in females in either the frontal cortex or the corpus callosum. NIST SRM 1650b females had reduced levels of GFAP immunostaining in the CA1 of the hippocampus than the controls (β = − 1.94, = SE =0.857, *p* = 0.047, *n* = 6). Females exposed to NIST SRM 1650b had higher group mean MBP immunostaining levels in the corpus callosum, although differences failed to reach statistical significance (β = 2.199, SE =0.857, *p* = 0.101, *n* = 6). There were no significant treatment-related differences for ventricle area in females.

NIST SRM 1650b males had significantly higher levels of GFAP immunostaining in the corpus callosum (β = 2.642, SE =1.188, *p* = 0.050, *n* = 6) and frontal cortex (β = 1.963, SE = 0.765, *p* = 0.028, *n* = 6) than controls, while there were no treatment-related differences in the hippocampus. There were no significant treatment-related differences in MBP immunostaining levels or ventricle area size in males.

### Body weights

**Fig. 4:**
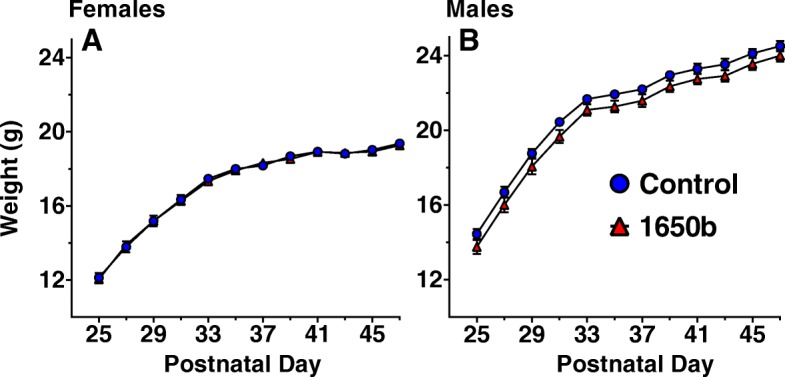
Juvenile and early-adult growth trajectories. Weights of females (**a**) and males (**b**) every other day after weaning (postnatal day 25). *n* = 16 mice/sex/treatment. Data are reported as group mean weight for each day ± SE

The body weight growth trajectories following weaning are shown for females (Fig. 4a) and males (Fig. 4b). Statistical analyses revealed no significant treatment-related differences in weight or growth rate in females during the post-weaning period. NIST SRM 1650b exposed males exhibited lower mean body weights throughout this period but, the differences failed to reach statistical significance from postnatal days 25–33, (β = − 0.378, SE = 0.213, *p* = 0.085, *n* = 16) or from postnatal days 35–47 (β = − 0.287, SE =0.184, *p* = 0.129, *n* = 16).

### Locomotor activity

**Fig. 5:**
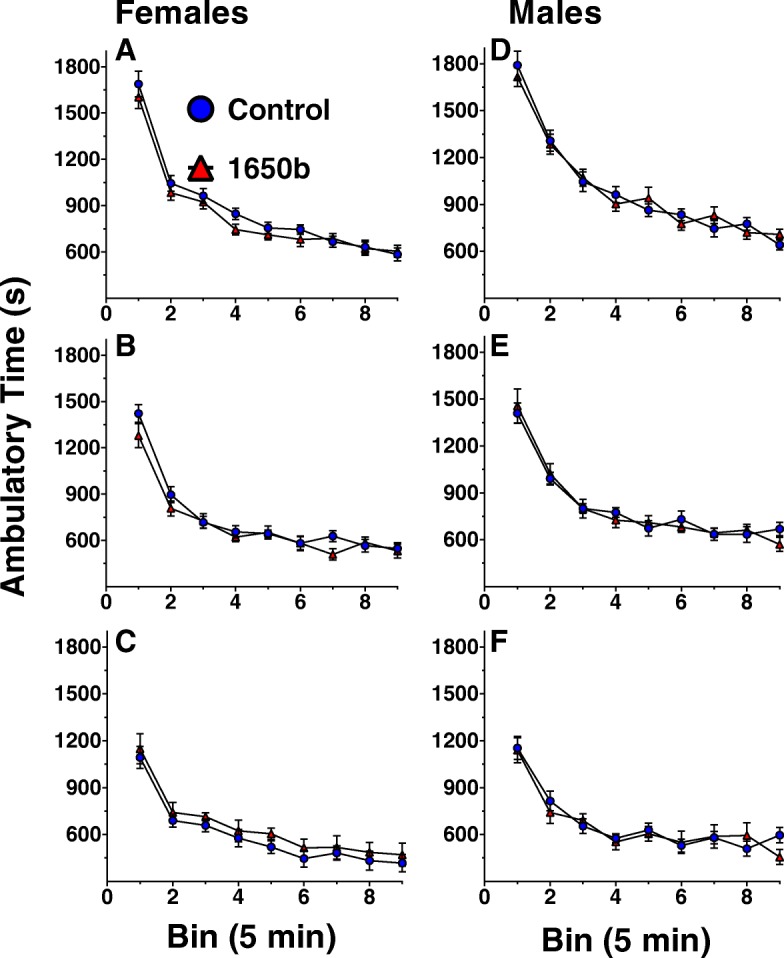
Locomotor Activity. Ambulatory times in 5 min epochs across three sessions for females (**a, b, c)** and males (**d, e, f**) across three 45-min sessions. Ambulatory movement was defined as successive breaks of multiple 2 × 2 defined photobeam virtual boxes within the chamber. *n* = 16 mice/sex/treatment. Data are reported as group mean ambulatory time for each bin ± SE

Ambulatory time of mice during the three locomotor sessions is shown for females (Fig. 5a-c) and males (Fig. 5d-f). Neither average ambulatory time nor activity habituation (slope) differed significantly different across exposure groups for either males or females in any session (*n* = 16).

### Fixed-interval 60s schedule

**Fig. 6:**
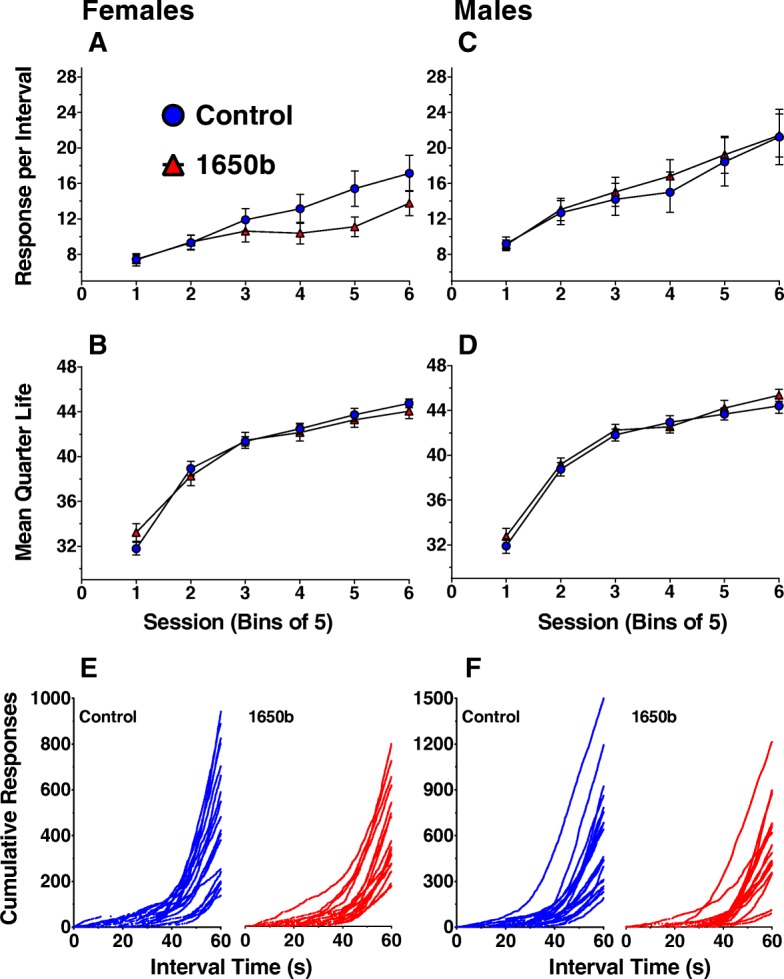
Fixed-interval 60s Schedule Behavioral Outcomes. Group mean responses per interval and mean quarter life for females (**a**, **b**) and males (**c, d**)**.** Data are reported as group mean responses per interval or mean quarter life for each bin of 5 sessions ± SE. A linear mixed-model was used to assess for NIST SRM 1650b-mediated differences with significance being defined as *p* ≤ 0.05. *n* = 16 mice/sex/treatment. Individual cumulative responses summed across the intervals in the final session for females (**e**) and males (**f**)

All mice learned food-rewarded lever pressing within two training sessions, without treatment-related differences in the number of sessions required for training. The responses per interval and the mean quarter life across 30 sessions on the fixed-interval (FI) 60s schedule are shown for females (Fig. 6a, b) and males (Fig. 6c, d).

Females exposed to NIST SRM 1650b exhibited a decreased learning slope for FI response rates over the 30 sessions but, the reduction did not attain statistical significance (β = − 0.087, SE = 0.046, *p* = 0.070, *n* = 16) and there were no treatment-related differences in average response rate across the 30 sessions (Fig. 6a). There were no significant overall treatment-related differences in mean quarter life for females across the 30 sessions (Fig. 6b). For males, there were no significant treatment-related differences in the learning slope or average across the 30 sessions for either average response rate (Fig. 6c) or mean quarter life (Fig. 6d). Readout of the individual cumulative records across the interval length within the final session are shown (Fig. 6e, f), with all mice in each treatment group displaying the classical “scallop” phenotype of the FI schedule.

## Discussion

Given the epidemiological evidence demonstrating an association between early-life exposure to traffic-derived pollution particulate and adverse neurodevelopmental outcomes, including ASD, selective attention deficits, and impulsivity, it is critical to provide clarity on the specific sources or constituents of particulate matter that could contribute to these phenotypes. Diesel particulate is considered to be a potential neurotoxic constituent of air pollution with previous studies demonstrating its neuroinflammatory potential [43–48]. The present study assessed the effects of neonatal inhalation exposure to re-suspended diesel exhaust particles on early life growth, as well as brain pathological measures and protracted learning deficits. The present study utilized a targeted exposure mass concentration, 100 μg/m^3^, for neonatal mice that was calculated via MPPD to be an equivalent pulmonary deposition dose to a young human child exposed to 13 μg/m^3^over 24 h. This dose has relevance as it is only slightly higher than the PM mass fraction of diesel particulate within the United States, which is estimated to be anywhere between 1 and 10 μg/m^3^ [19, 55–57]. Overall only minor differences between exposed and control mice were observed, with a distinct lack of protracted behavioral and learning alterations following diesel exhaust particulate matter exposure.

The increase in GFAP immunoreactivity levels in the treated males frontal cortex and corpus callosum could potentially indicate an inflammatory response to the NIST SRM 1650b exposure which, has been shown in previous ambient developmental exposures to fine and ultrafine particles [8, 9, 16]. This also supports previous evidence diesel particle exposure can lead to inflammatory outcomes within the CNS [43, 44, 47, 58]. The decrease of GFAP immunoreactivity in the CA1 of the NIST SRM 1650b females’ hippocampus was unexpected and could potentially result from a loss of astrocytes, a reduction of astrocyte processes, and/or growth within the region. Interestingly, a previous study found exposure to ultrafine particulate matter in young female C57BL/6 mice led to neurite atrophy and a decrease in glutamate receptor expression, a receptor found prominently on astrocytes within the CA1 region of the hippocampus [59]. Despite the pathological changes in males and females immediately following exposure, it seems unlikely these produce long-term CNS functional changes, as there were no significant treatment-differences on the locomotor assay or learning phenotypes on the fixed interval schedule. The potential exists for other behavioral domains to be affected, such as short-term memory assessed via a paradigm like novel object recognition, a question requiring further consideration.

Despite the suggestive inflammatory results from NIST SRM 1650b, there was no observed white matter pathology within the corpus callosum or indication of ventriculomegaly, a condition that typical co-occurs with atrophy of the corpus callosum [60]. These pathological conditions are of interest as they are often seen in children with ASD [61–63] and ADHD [64, 65], the same disorders linked to adverse neurodevelopmental outcomes from early-life exposure to pollution particulate [14, 15, 66]. Additionally, there is some direct preliminary evidence children exposed to high concentrations of ambient fine particles in Mexico City had abnormal white matter pathology and altered fMRI readouts [67–69]. Complementing the human studies, ambient ultrafine particulate matter in mice, at an exposure equivalent timeframe to the presented study, has shown deficiencies in white matter within the corpus callosum, as well as increased ventriculomegaly [8, 10]. One limitation of the present study is the pathology was explored only 24 h after the last day of exposure, based on prior studies that found traffic-related ultrafine particulate induced pathological effects in that time frame [8, 9]. There is the capacity for “silent” neurotoxicity in which an adverse series of underlying key molecular events is initiated during exposure but the final pathological changes only manifest in the adolescent or adult following the completion of development. However the lack of significant functional learning phenotypes in adulthood suggests no detrimental pathology developed. The exact pathways by which pollution particulate produces white matter pathology is still unclear but, the data presented in this study suggests diesel particulate exposure alone is not a sufficient contributor.

Although it failed to reach statistical significance, there was a noticeable sustained decrease in body weight in males exposed to NIST SRM 1650b during the juvenile/adolescent period, even though their growth rates were equivalent to controls. The research investigating the effects of developmental exposure to diesel exhaust particles on body weight and growth is mixed. Some studies utilizing prenatal models of diesel exhaust exposure have shown a reduction in body weight following weaning [70], increased body weight in early-adulthood [71], and no change in body weight in either adolescence or adulthood [72, 73]. The exact pathways to these weight alterations is unclear, one reason could be appetitive, with diesel exhaust shown to increase the release of corticosterone in the presence adrenocorticotropic hormone in males [74], which is a known inducer of body weight loss [75]. However, there was no increase or decrease in response rates with NIST SRM 1650b treated males on the FI schedule despite substantial food restriction which, would have been expected if there was a motivation or appetitive influence from treatment. An alternative explanation is diesel particles directly influences metabolism with some studies showing diesel particulate influences fatty acid metabolism and uptake within the liver [76, 77]. Further clarification on the early-life effects of diesel particulate matter on metabolism and body mass index may be warranted.

Interestingly, though not statistically significant, females exposed to NIST SRM 1650b had a decrease in response rate learning on the FI schedule of reward over time, but no differences in recognizing or responding appropriately to the temporal aspect of the schedule. A decrease in response rates over time could be linked to several underlying behavioral mechanisms, including hypo-motivation, hypoactivity, and/or attention impairments. However, the locomotor assay did not suggest any gross activity-level treatment-related differences and attention impairments is unlikely given the NIST SRM 1650b females still recognized the temporal aspect of the schedule as demonstrated through mean quarter life values equivalent to controls. This study is not the first to show a lack of significant behavioral outcomes with regards to diesel particle exposure. In a previous study, gestational inhalation exposure to re-suspended NIST SRM 2975 diesel particulate material did not produce any learning deficits in the offspring as assessed using a Morris water maze paradigm [70]. The data presented in this study suggest the diesel particulate alone, even during a critical period of development, may not be sufficient to induce substantial protracted cognitive dysfunction.

One of the primary limitations of the NIST SRM 1650b is it does not contain volatile and semi-volatile diesel species generated from combustion and its interactions with particulates cannot be examined with aged material and can only be captured with freshly generated diesel or with real-time ambient exposures. Diesel exhaust studies that have shown adverse neurotoxic outcomes in adult [45, 58] and development exposures [78] utilized freshly-generated engine emissions in which the setup includes volatile organic compounds (VOCs), and gas components [79]. Some of the gas components within those studies, ozone [80, 81], carbon monoxide [82], and nitrogen oxides [83, 84] all have known potential to influence the CNS. Furthermore, the presence of ozone and VOCs can lead to the formation of secondary organic aerosols (SOAs) that may be important contributors to the neurotoxic effects of diesel emissions [85, 86]. Indeed, the presence of diesel-derived SOAs have been shown to enhance the neurotoxic effects of the diesel particulate component in an adult exposure model [87]. Complementing the diesel studies, studies exploring ambient traffic-related ultrafine particle exposures with an equivalent time frame as the present study [7–9, 88], used an exposure system that maintains the presence of ambient gaseous and semi-volatile components [89] that also may have been neurotoxic contributors. The null results from the present study, which utilized a limited non-volatile diesel particulate matter material, may suggest it is necessary to include additional components, VOCs, semi-volatile and gas components from diesel emissions to appropriately evaluate neurotoxic potential.

Using NIST SRMs for toxicological studies has other limitations, including the age, the need to re-suspend in water, and to ultra-sonicate the mixture for aerosolization, all of which can alter the physiochemical and potentially toxic properties that would otherwise come from combustion. Although the median particle size was close to the ultrafine size, the substantial presence of larger particles likely comes from aggregation and agglomeration following the original combustion reaction, as well as the subsequent aerosolization. Tween 80 was utilized to prevent aggregation of the particles within the solution but, was not considered a concern for particle uptake as it has been previously shown to have little effect on re-suspended nanomaterials with regards to particle size, polydispersion index, and zeta potential [90]. Although Tween 80 has been shown to enhance nanomaterial uptake into the nasal epithelium [91] and blood-brain barrier membranes [92, 93], it is worth noting those particles were placed in solutions with a concentration ≥ 0.5% Tween 80, about 1000x the concentration of the SRM 1650b solution. Aging of diesel particulate matter has been shown to alter its oxidative potential which, could also influence its capacity to induce neurotoxic effects [94]. The insoluble fraction of NIST SRM 1650b, which could contain harmful organic and metal content, can settle across the exposure period and thus be lost in the inhalation exposure. Despite these limitations, the advantages of using NIST SRM 1650b are it is a well-characterized diesel material with an exposure method that can be reproduced.

Other limitations that come with the study include the use of the MPPD program to calculate equivalent doses between mice neonates and young human children. Although the majority of the neonatal mouse physiological parameters were based on relevant background data, certain parameters of the MPPD program including the segmental anatomy of the lung and total number of terminal airways are based off static default mouse adult data. The neonatal mouse lung, especially the distal alveolar regions, is rapidly developing during the exposure time period [95–97] making equivalent alveolar deposition estimations difficult. Additionally with regards to the CNS, it is unclear if estimations of alveolar deposition of diesel are the best dosing parameter given the indirect pathway by which particles deposited in the nasal epithelium can also access the CNS. Future studies that can delineate between the neurotoxic effects of UFP deposited in the pulmonary system vs UFP deposited in the nasal epithelium may provide clarity on this issue.

With regards to the mice exposure conditions, although using internal litter controls has advantages in eliminating litter-specific/maternal behavioral effects, it does create a scenario for diesel particulate that settled on the fur of the exposed pups to cross-contaminate over to littermates as well as lead to indirect dam exposure from maternal grooming behavior. Another important concern is pup thermoregulation in the inhalation chamber, which was addressed by ensuring every pup had at least one littermate in the inhalation cages with natural pup huddling behavior shown to be an effective means to regulate C57BL/6 pup body temperatures [98, 99]. However some heat loss in separated pups is expected at the exposure temperatures even with accompanying littermates [98] and is a potential extraneous factor experienced by controls and exposed mice. Lastly, although removing the pups from the dams for exposures was necessary to evaluate the direct effects of NIST SRM 1650b inhalation on pup neurodevelopment, repeated maternal separation is considered a stress-inducing factor [100, 101] and can alter physiological responses to subsequent stressors [102, 103]. Consideration of repeated maternal separation as an extraneous factor that could override the effects of particle exposure is warranted though studies investigating repeated maternal separation’s protracted effects on offspring behavior have mixed findings with null [104–106] and adverse [107–109] results.

## Conclusions

Identifying the underlying critical components of pollution particulate that contribute to adverse neurodevelopmental phenotypes could have direct relevance to creating a focused framework for the regulation of air pollution levels. The present study was done to determine whether neonatal diesel particle exposure alone could induce adverse pathology and persistent behavioral dysfunction. While neonatal exposure to NIST SRM 1650b induced some pathological changes in males suggestive of inflammation, it had no substantial protracted influence on either locomotor activity or learning alterations as assessed using FI performance in either males or females. As developmental exposure to isolated diesel particulate alone was not sufficient to induce protracted adverse outcomes, further clarity on the toxic contribution of specific gaseous, semi-volatile, and volatile components within diesel emissions may be necessary to appropriately evaluate neurodevelopmental risk.

## Methods

### Breeding

Eight-week-old male and female C57BL/6 mice were purchased from Jackson Laboratories (Bar Harbor, ME) and allowed to acclimate in the vivarium housing room for one week prior to breeding. All mice in this study were housed under a 12-h reversed light/dark cycle and temperature maintained at ~ 22 °C. Three days prior to pairing, dirty male bedding was added to the female cages to synchronize estrous cycles (Whitten effect) and increase the likelihood of impregnation. Monogamous pairs of mice were bred for three days, males were then removed and dams remained singly housed with litters until weaning. Neonatal mice were separated from the dams for the whole-body NIST SRM 1650b inhalation exposures (the dams were not directly exposed) which, occurred from postnatal days (PNDs) 4–7 and PNDs 10–13 for 4 h/day for 4 days/week between 1000 and 1400 h. During these exposures, the mice pups were housed in small mesh chambers with 2–4 pups per chamber. The mice pups were returned to the original dam upon completion of each exposure session. Eighteen dams were used for the study with the sample size, *n,* referring to the total number of litters utilized for the endpoint. To eliminate litter-specific effects, each litter was split internally into control and treated pups, half the pups within a litter would be exposed to HEPA-filtered air and the other half exposed to NIST SRM 1650b. The range of litter sizes was 5–9 pups.

### Exposure

Exposure conditions are based on modeling calculations (Additional file 1) [110] to produce equivalent particle deposition per alveolar surface area in the mice pups with a 4 h exposure to that of a 3 month human infant exposed to a diesel mass concentration of 13 μg/m^3^ over 24 h. NIST Standard Reference Material 1650b (http://www.nist.gov/srm) was suspended in a solution of 5 μl/L TWEEN80 (Millipore Sigma, St. Louis, MO) in 18 M Ω water. The NIST SRM 1650b powder was mixed in 10 mg/mL of solution ratio and sonicated with a probe sonicator (Sonics & Materials Inc., Newtown, CT) at 750 watts, 20 kHz frequency, for three, ten second bursts. 15 mL of this solution was put into an ultrasonic nebulizer (Model Ultrasonic2000, Nouvag Dental and Medical Equipment, Goldach, Switzerland) to generate the diesel mist. Clean dry air (500–1000 mL/min) was passed through the nebulizer at a frequency of 2.4 MHz to produce the diesel mist which, was then passed through a heated drying tube and cold trap to remove moisture. The resulting dry aerosol was mixed with diluting air and entrained into a 30 L stainless steel-reinforced Lexan exposure chamber housing the neonatal mice at a rate of 25–30 L/min. A peristaltic pump (Masterflex, Cole Palmer Inc., Mount Vernon, IL) was used to maintain the diesel solution level in the nebulizer for the duration of the exposure session. The ambient temperature in the inhalation chambers ranged from 23 to 25 °C and humidity was maintained at 40–50% for controls and exposed mice. Real-time chamber exposure measurements were continuously performed through the 4 h exposure using a condensation particle counter set up downstream of the inhalation chamber (CPC, Model 3022A TSI Inc., Shoreview, MN). The particles were sized in a single five minute run at the end of the 4 h exposure using an electrostatic classifier (SMPS Model 3071, TSI Inc., Shoreview, MN). Filters were periodically collected to determine gravimetric exposure concentration by mass. The target value was an overall average of 100 μg/m^3^. For imaging, NIST SRM 1650b particles were collected via electrostatic precipitation on carbon coated copper grids (CF-200CU, Electron Microscopy Sciences) which, were imaged using a Hitachi 7650 Analytical TEM with an Erlangshen 11 megapixel digital camera and Gatan software.

### Pathology

On PND 14, mice were euthanized by rapid decapitation in the absence of anesthesia to assess the immediate effect of NIST SRM 1650b on brain pathological outcomes. Brains were extracted and placed in 4% paraformaldehyde for 24 h, then into 30% sucrose until they sank. The brains were sectioned on a freezing microtome (Microm HM 440 E; GMI Inc., Ramsey, MN) at 40-μm thickness in cryoprotectant (30% sucrose, 30% ethylene glycol in 0.1 M phosphate buffer) and stored at − 4 °F (− 20 °C) until immunostaining. Every sixth section was stained with glial fibrillary acidic protein (GFAP) and myelin basic protein (MBP) to assess, respectively, activation of astrocytes and potential alterations in the extent of white matter. Briefly, the brain sections were washed of cryoprotectant and placed into primary antibody solutions for GFAP (AB5804, 1:3000 dilution; Millepore, Billerica, MA) or MBP (MAB386, 1:1000; Millepore, Billerica, MA) for 24 h. For GFAP, the tissue was then placed into a biotinylated anti-rabbit IgG antibody solution (BA-1000, 1:200 dilution; Vector Labs, Burlingame, CA) and for MBP, a biotinylated anti-rat, mouse-absorbed, IgG antibody (BA-9401, 1:200 dilution; Vector Labs, Burlingame, CA) for 1 h, and the stain was visualized using DAB diaminobenzidine hydrochloride (D0426, SIGMA FAST DAB with metal enhancer, Sigma Aldrich, St. Louis, Mo., USA). Immunolabeled tissue was mounted onto Superfrost Plus micro slides (48311–703; VWR, Radnor, PA) and coverslipped using Cytoseal 60 (23–244,257; Fisher Scientific, Pittsburg, PA).

Slide-mounted tissue sections were visualized on an Olympus BX41 microscope (Olympus America, Inc., Central Valley, Pennsylvania) mounted with an MBF CX9000 camera (MBF, Villiston, Vermont) for GFAP and MBP image capture. Lateral ventricle area tracings were performed using Neurolucida software (MBF, Williston, Vermont) with three consecutive sections per subject used. To quantify MBP and GFAP immunostaining intensity, images were captured at 40x at the same exposure and brightness settings, and analyzed by optical densitometry using NIH ImageJ software. Optical density was deduced from mean grayscale analysis of the region of interest (ROI) standardized to a negatively stained region within the same section to normalize background grayscale. Three measurements were taken on three separate sections per brain per ROI. A murine anatomical brain atlas was utilized in conjunction with identification of anatomical landmarks to ensure the sections of homologous bregma were analyzed [111]. The ROIs for MBP and GFAP immunostaining in the corpus callosum and frontal cortex were bregma 1.32–2.04 mm and GFAP immunostaining in the hippocampus at bregma 1.74–2.74 mm. The results are expressed as the difference value between the background greyscale value and the positively-stained region, with higher values demonstrating increased immunoreactivity. Density and ventricular area analyses were carried out blinded to treatment condition.

### Locomotor assay

Spontaneous locomotor activity was assessed in photobeam chambers equipped with a transparent acrylic arena with a 48-channel infrared source, detector, and controller (Med Associates, St. Albans, VT). Locomotor behavior was explored prior to the start of FI schedule-controlled behavior training (PND 60). Three 45-min sessions occurred on three consecutive days with the primary endpoint, ambulatory time, collected in 5 min epochs. Ambulatory time was defined as the cumulative time in which there were successive breaks of 2 × 2 photobeam virtual boxes within the chamber. Habituation and average ambulatory activity in each session was assessed.

### Operant behavior apparatus and fixed interval schedule

Food restriction followed locomotor assessment. To enhance and normalize motivation for a food-reinforcement, mice were placed on a food-restricted schedule for 3 days immediately prior to initiation of operant training, to reach 85% of ad libitum weight. Mice were maintained at 85% ad libitum body weight throughout the operant training schedule. Behavioral testing was conducted in operant chambers (Med Associates, St. Albans, VT) housed in sound-attenuating cabinets equipped with white noise and fans for ventilation. Three levers were located horizontally across the back wall of the chamber, with a pellet dispenser for reinforcer delivery on the front (opposite) wall. Mice were initially trained to press a lever for food reward using a variable time 60 s fixed ratio 1 schedule (VT60FR1), in which a reinforcer (20 mg food pellet) was delivered simultaneously with a light and sound cue on average every 60 s independently of behavior; a response on the designated correct lever during this period would also trigger the light and sound cue, and provide reinforcement delivery. Following ten correct lever press responses or a total of 20 min on the VT60 component, the schedule was changed to a fixed ratio 1 schedule that required a lever press on the designated correct lever for each food delivery until 90 reinforcers had been delivered. After lever press training was completed in all mice, the schedule was shifted to a 60 s FI schedule (FI60), in which mice had to complete 30 intervals per day across 30 consecutive days. On the FI schedule, the first lever press response on the designated correct lever after completion of a 60 s interval produced food delivery and initiated the next 60 s interval. Responses during the interval itself had no explicit consequence, i.e., were not consequated.

Measures of FI performance included average response per interval and mean quarter life. Quarter life, the latency from the onset of an interval to the time at which the first one quarter of the responses in the interval occurred, was used to assess temporal control. Initially, performance on the FI schedule is characterized by uniform responding throughout the interval. However, over sessions, as temporal control is established, pausing begins to follow reinforcement delivery and maximal responding shifts to later in the interval, with quarter life values thus initially increasing significantly over early sessions, followed by more gradual but continual increases as behavior stabilizes. To prevent inappropriate skewing of FI performance measures due to high initial behavioral variation when starting the fixed interval schedule, the first three intervals of each session were not included in the overall analyses.

### Statistical analyses

All behavioral and pathological analyses were stratified by sex. Body weight, as well as ambulatory time, and fixed-interval data were analyzed using a mixed model approach as previously described [88]. A random intercept and random slope model was used to capture individual-level variability and two fixed variables were used capture group-level variation, slope and NIST SRM 1650b exposure. The intercept component was centered and used a means of exploring the average values across the days, while a slope was used to define the linear function of growth across the days. As the body weight and mean quarter life values produced non-linear growth curves, a piecewise approach was utilized in which the curve was broken into two linear segments. For the weights, a slope was fitted for P25-P33 and another for P35-P47 and for the mean quarter life, a slope was fit for sessions 1–12, and sessions 13–30.

The pathological measures were analyzed using a random-intercept only model in which the average optical densitometry (MBP, GFAP) or area (ventricles) across the three ROIs within each subject was evaluated. All analyses were conducted using the mixed-model function within JMP13 Pro (SAS Institute Inc., Cary, NC). Estimates (β), standard errors (SE), and *p*-values are reported. The a priori statistical significance criterion was α ≤ 0.05, though p-values ≤0.1 are also noted in the results.

## Additional files

Additional file 1: Multiple-Path Particle Dosimetry. (DOCX 21 kb)

Additional file 2: Raw Dataset. (XLSX 1310.72 kb)

## Abbreviations

ASD: Autism spectrum disorder
CMD: Count median diameter
CNS: Central nervous system
FI: Fixed interval
GFAP: Glial fibrillary-acidic protein
LDH: Lactate dehydrognease
MBP: Myelin basic protein
NIST SRM: National Institute of Standards and Technology Standard Reference Material
PAH: Polycyclic aromatic hydrocarbons
PM: Particulate matter
TEM: Transmission electron microscopy
UFP: Ultrafine Particles
